# Statin Therapy and Clinical Outcomes in Metabolic Dysfunction-Associated Steatotic Liver Disease: A Systematic Review and Meta-Analysis

**DOI:** 10.7759/cureus.104139

**Published:** 2026-02-23

**Authors:** Iman Gillani, Muhammad Muneeb Ullah, Anastasia Postoev, Ashutosh Sharma, Rahman Hameed Mohammed Abdul, Sonalben Chaudhary, Himashi Gunaratne, Danish Allahwala

**Affiliations:** 1 Medical Education, Shaikh Khalifa Bin Zayed Al Nahyan Medical & Dental College, Lahore, PAK; 2 Medicine and Surgery, Rashid Latif Medical College, Lahore, PAK; 3 Internal Medicine, Caribbean Medical University, Willemstad, CUW; 4 Medicine, Kathmandu Medical College and Teaching Hospital, Kathmandu, NPL; 5 Gastroenterology and Hepatology, Royal Derby Hospital, Derby, GBR; 6 Internal Medicine, Zydus Sitapur Hospital, Sitapur, IND; 7 Medicine, University of Colombo, Colombo, LKA; 8 Nephrology, Kathmandu Medical College and Teaching Hospital, Kathmandu, NPL

**Keywords:** hepatocellular carcinoma, meta-analysis, metabolic dysfunction-associated steatotic liver disease, mortality, statins

## Abstract

Beyond their lipid-lowering effects, statins exert pleiotropic properties, including anti-inflammatory, antioxidant, and antifibrotic actions, which may confer therapeutic benefits in patients with metabolic dysfunction-associated steatotic liver disease (MASLD). This systematic review and meta-analysis aimed to evaluate the impact of statin therapy on clinical outcomes in adults with MASLD. A comprehensive literature search was conducted across PubMed, Embase, Cochrane CENTRAL, Web of Science, and Scopus from inception through January 10, 2026. Observational studies and randomized controlled trials comparing statin therapy with placebo or no treatment in adults with MASLD were eligible for inclusion. Eight observational studies met the inclusion criteria and were included in the quantitative synthesis. The primary outcomes were hepatocellular carcinoma incidence, all-cause mortality, and liver-related mortality. Statin use was associated with a significantly lower risk of hepatocellular carcinoma (pooled risk ratio (RR) 0.56, 95% CI 0.45-0.71), all-cause mortality (pooled RR 0.81, 95% CI 0.72-0.91), and liver-related mortality (pooled RR 0.54, 95% CI 0.37-0.79). Moderate to substantial heterogeneity was observed across studies. These findings suggest that statin therapy may confer meaningful hepatic and survival benefits in patients with MASLD, supporting their proactive use, particularly given their concomitant cardiovascular advantages. Nonetheless, the observational nature of the included studies underscores the need for large-scale randomized controlled trials to establish causality and define optimal treatment strategies.

## Introduction and background

Previously termed non-alcoholic fatty liver disease (NAFLD), metabolic dysfunction-associated steatotic liver disease (MASLD) has become the most common chronic liver disorder globally, impacting roughly 30% of adults worldwide [1]. This condition involves fat accumulation in the liver without substantial alcohol intake and demonstrates strong links to obesity, type 2 diabetes mellitus, dyslipidemia, and cardiovascular disease [2]. The clinical presentation varies from isolated steatosis to metabolic dysfunction-associated steatohepatitis (MASH), with potential advancement to cirrhosis, hepatocellular carcinoma, and mortality from liver complications [3]. With the worldwide increase in metabolic syndrome prevalence, MASLD has emerged as a primary indication for liver transplantation and poses a considerable public health challenge [4].

Despite the growing clinical importance of MASLD, there remains no universally approved pharmacological therapy specifically targeting this condition. Current management strategies primarily focus on lifestyle modifications, including weight loss, dietary changes, and increased physical activity [5]. However, the high rates of treatment non-adherence and difficulty in achieving sustained weight loss have created an urgent need for effective pharmacological interventions. Given the strong association between MASLD and cardiovascular risk factors, medications that address both hepatic and cardiometabolic abnormalities are of particular interest [6].

Statins, which inhibit 3-hydroxy-3-methylglutaryl coenzyme A (HMG-CoA) reductase, are commonly prescribed medications for lowering lipids with proven cardiovascular advantages. In addition to reducing lipid levels, statins exhibit multiple beneficial effects, including anti-inflammatory, antioxidant, and antifibrotic properties, which could prove advantageous in treating MASLD [7]. Laboratory research has shown that statins may decrease fat accumulation in the liver, reduce inflammation, and lessen fibrosis through various pathways, including altering lipid processing, decreasing oxidative stress, and preventing the activation of hepatic stellate cells [8]. Additionally, observational research has indicated possible connections between statin therapy and lower risks of MASLD advancement and hepatocellular carcinoma development [9].

However, concerns regarding statin safety in patients with liver disease have historically limited their use in this population, despite evidence suggesting that statins are generally safe and well-tolerated in patients with chronic liver disease, including those with MASLD [10]. Several randomized controlled trials (RCTs) have investigated the effects of statins on liver enzymes, hepatic steatosis, fibrosis markers, and histological outcomes in MASLD patients, but individual studies have yielded inconsistent results with varying methodologies and outcome measures [11].

Given the growing body of evidence from multiple studies and the clinical need to identify effective therapeutic strategies for MASLD, a comprehensive synthesis of the available data is warranted. Previous meta-analysis focused on biomarkers, we need to assess the impact on clinical outcomes [12]. Accordingly, this meta-analysis aims to systematically evaluate the effects of statin therapy on clinical outcomes in patients with MASLD, thereby providing a robust evidence base to inform clinical decision-making and guide future research.

## Review

Methodology

This systematic review and meta-analysis was conducted as per the guidance of Preferred Reporting of Systematic Review and Meta-analysis (PRISMA) [13]. The protocol for this study was registered with International Prospective Register of Systematic Reviews (PROSPERO; CRD42044548832).

Literature Search

An extensive systematic search was conducted in several electronic databases such as PubMed/MEDLINE, Embase, Cochrane Central Register of Controlled Trials (CENTRAL), Web of Science, and Scopus covering the period from their establishment to 10 January 2026. The search approach included Medical Subject Headings (MeSH) and unrestricted text terms associated with the primary concepts of: (1) metabolic dysfunction-related steatotic liver disease (MASLD), non-alcoholic fatty liver disease (NAFLD), and non-alcoholic steatohepatitis (NASH); (2) statins encompassing both generic terms and specific agents (atorvastatin, simvastatin, rosuvastatin, pravastatin, fluvastatin, lovastatin, pitavastatin); and (3) randomized controlled trials. Boolean logic operators (AND, OR) were applied to combine these search terms effectively. The citation lists of retrieved publications, pertinent systematic reviews, and meta-analyses were thoroughly examined to discover additional studies that may have been missed in the electronic database search. Conference abstracts from significant hepatology and endocrinology conferences (American Association for the Study of Liver Diseases (AASLD), European Association for the Study of the Liver (EASL), and the Endocrine Society) from the preceding five years were also reviewed. No restrictions based on language were applied, and publications in languages other than English were translated as needed. Clinical trial registries such as ClinicalTrials.gov and the WHO International Clinical Trials Registry Platform were explored to find unpublished or active studies. Two authors independently conducted the search. Any differences between the two authors were settled through discussion.

Study Selection

The collected records were uploaded to Covidence systematic review software (Veritas Health Innovation, Melbourne, Australia), which automatically identified and eliminated duplicate entries. Subsequently, two independent reviewers manually examined titles and abstracts using pre-established eligibility criteria during the preliminary screening phase. Studies that did not satisfy the inclusion requirements were removed at this point. The complete manuscripts of studies that appeared potentially relevant were then retrieved and evaluated independently by the same pair of reviewers. When reviewers disagreed during either screening phase, differences were addressed through discussion, and if agreement could not be achieved, a third reviewer was brought in to make the final decision.

Studies were incorporated into the review if they fulfilled these requirements: (1) they were RCTs or observational designs (case-control or cohort studies) that examined statin treatment compared to placebo or no statin intervention; (2) they involved participants who were 18 years of age or older with MASLD/NAFLD verified through imaging, histology, or validated diagnostic methods; and (3) they presented at least one pertinent outcome metric such as hepatic carcinoma, overall mortality, or liver-related mortality. Studies comprising heterogeneous populations were considered eligible when MASLD-specific subgroup data were reported separately, enabling extraction of disease-specific estimates.

Studies were omitted if they: (1) enrolled patients with alternative etiologies of chronic liver disease (viral hepatitis, alcohol-related liver disease, autoimmune hepatitis, primary biliary cholangitis, hemochromatosis, Wilson's disease); (2) did not include a control group; (4) were duplicate publications describing the same patient population; or (5) lacked adequate data for meta-analysis even after attempting to contact the authors.

Data Extraction

Two independent reviewers used a standardized Microsoft Excel (Microsoft Corp., Redmond, WA, USA) data extraction form that had been pilot-tested to gather pertinent details from all studies in the review. Any differences in the extracted information were reconciled through discussion and checked against the source publications. The researchers systematically collected the following information: study characteristics (primary author, year of publication, country of origin, study methodology, and sample size), participant demographics (age and sex), and results. For studies measuring outcomes at several timepoints, the data from the final follow-up assessment were used. When studies included multiple treatment groups, only the information comparing statin therapy to control conditions was extracted.

Quality Assessment

The methodological quality and risk of bias of included randomized controlled trials were independently evaluated by two reviewers using the Cochrane Risk of Bias tool version 2 (RoB 2) [14]. For observational studies, the Newcastle-Ottawa scale (NOS) was used [15].

Statistical Analysis Plan

Statistical analyses were conducted using Review Manager (RevMan version 5.4, The Cochrane Collaboration, London, England, UK). For dichotomous outcomes, including the incidence of hepatocellular carcinoma, all-cause mortality, and liver-related mortality, pooled risk ratios (RRs) with 95% confidence intervals (CIs) were calculated.

Effect measures were reported as hazard ratios (HRs), RRs, or odds ratios (ORs). RRs were prespecified as the common metric for pooling. HRs were considered equivalent to RRs and were therefore pooled directly with RRs using the generic inverse-variance method. For studies reporting ORs, RRs were calculated from the raw event data (number of events and total participants in each group) when available, thereby ensuring consistency in the summary measure. All effect estimates were transformed to the natural logarithmic scale, and corresponding standard errors were derived from the reported 95% confidence intervals. Pooled estimates were calculated using a random-effects model to account for anticipated between-study heterogeneity.

Statistical heterogeneity was evaluated using Cochran’s Q test and the I² statistic, with I² values of 25%, 50%, and 75% representing low, moderate, and high heterogeneity, respectively. A P value <0.10 for the Q test was considered indicative of statistically significant heterogeneity. Forest plots were generated to illustrate individual study estimates and pooled effect sizes with corresponding 95% CIs. Statistical significance for all outcomes was defined as a two-sided P-value <0.05. As the number of included studies was less than 10, we were unable to perform publication bias.

Results

**Figure 1:**
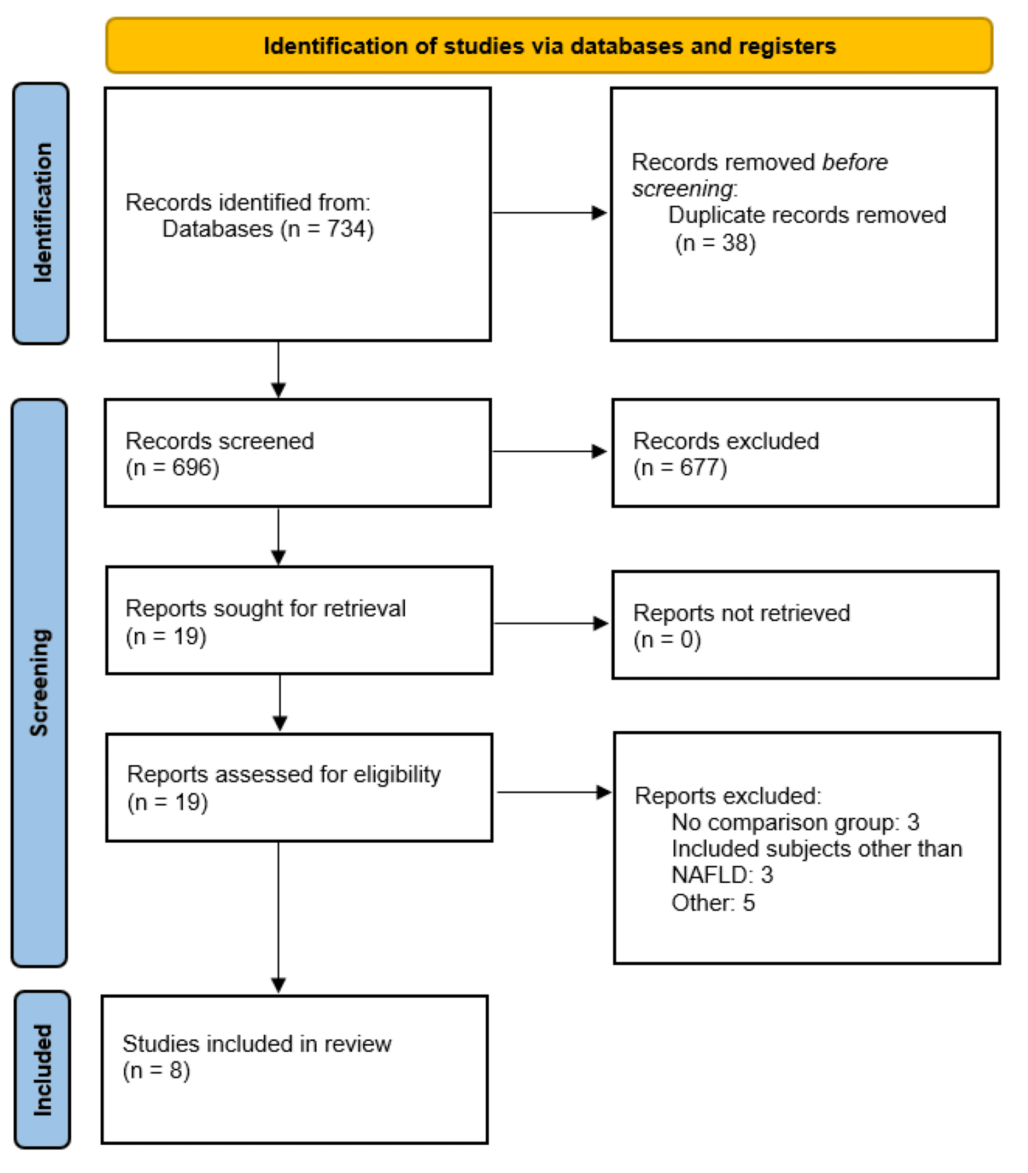
PRISMA flowchart (study selection process) PRISMA: Preferred Reporting Items for Systematic Reviews and Meta-Analyses.

A total of 734 records were identified through database searches. After removing duplicates, 696 studies remained for title and abstract screening, of which 19 were deemed eligible for full-text review. Ultimately, eight studies met the inclusion criteria and were included in the meta-analysis. The study selection process is depicted in the PRISMA flow diagram (Figure 1).

The characteristics of the included studies are summarized in Table 1.

**Table 1:** Characteristics of the included studies (n=8)

Author	Year	Design	Region	Sample size
German et al. [16]	2020	Case-control	United States	102
Lee et al. [17]	2017	Retrospective cohort	Taiwan	18000
Ng et al. [18]	2022	Retrospective cohort	Multi-national	12538
Pinyopornpanish et al. [19]	2021	Retrospective cohort	United States	1072
Yang et al. [20]	2025	Retrospective cohort	United Kingdom	224825
Yun et al. [21]	2025	Retrospective cohort	Korea	516575
Zhou et al. [22]	2024	Retrospective cohort	United States	7988
Zou et al. [23]	2023	Retrospective cohort	United States	272431

All included studies were observational in design, with the majority conducted in the United States. The quality assessment of the included studies is presented in Table 2.

**Table 2:** Quality assessment of the included studies using the Newcastle-Ottawa Scale

Author	Selection	Comparison	Assessment	Overall
German et al. [16]	2	1	2	Fair
Lee et al. [17]	3	2	2	Good
Ng et al. [18]	3	2	2	Good
Pinyopornpanish et al. [19]	3	2	3	Good
Yang et al. [20]	4	2	2	Good
Yun et al. [21]	4	2	3	Good
Zhou et al. [22]	3	2	2	Good
Zou et al. [23]	4	2	2	Good

Majority of the included studies were of good quality (seven out of eight), so they are less likely to affect the results.

Hepatocellular Carcinoma (HCC)

**Figure 2:**
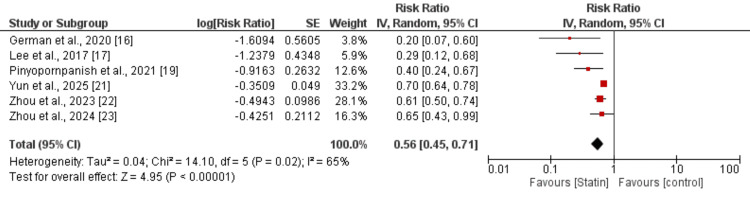
Effect of statin on the risk of developing hepatocellular carcinoma (HCC) [16,17,19,21-23]

Six studies were included in the meta-analysis comparing the risk of HCC between statin users and non-statin users. Overall, statin use was associated with a significantly reduced risk of HCC compared with non-statin use (pooled RR =0.56, 95% CI: 0.45-0.71; P<0.00001) as shown in Figure 2.

Moderate heterogeneity was observed among the included studies (I² = 65%). The direction of effect consistently favored statin users across all included studies. Table 3 shows the sensitivity analysis results. 

**Table 3:** Sensitivity analysis in hepatocellular carcinoma (HCC) RR: Risk ratio; CI: Confidence interval.

Author	RR (95% CI)	I-Square
German et al. [16]	0.60 (0.49 to 0.73)	59%
Lee et al. [17]	0.60 (0.48 to 0.74)	62%
Ng et al. [18]	0.60 (0.48 to 0.75)	61%
Pinyopornpanish et al. [19]	0.49 (0.35 to 0.67)	53%
Zhou et al. [22]	0.49 (0.34 to 0.72)	70%
Zou et al. [23]	0.53 (0.40 to 0.70)	72%

Sensitivity analysis for HCC outcome showed consistent risk reduction across all included studies, with pooled effect estimates ranging from RR 0.49 to 0.60 and moderate heterogeneity (I²: 53%-72%). These findings confirm the robustness and stability of the protective association for HCC.

All-Cause Mortality

**Figure 3:**
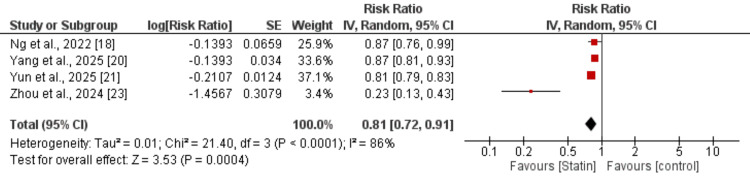
Effect of statin on the risk of death [18,20,21,23]

Four studies were included in the meta-analysis evaluating the association between statin use and the risk of mortality compared with non-statin use. Statin use was associated with a statistically significant reduction in the risk of mortality (pooled RR=0.81, 95% CI: 0.72-0.91; P=0.0004) as shown in Figure 3.

Substantial heterogeneity was observed across studies. Despite the high heterogeneity, all included studies demonstrated a direction of effect favoring statin users over non-statin users.

Liver-Related Death

**Figure 4:**
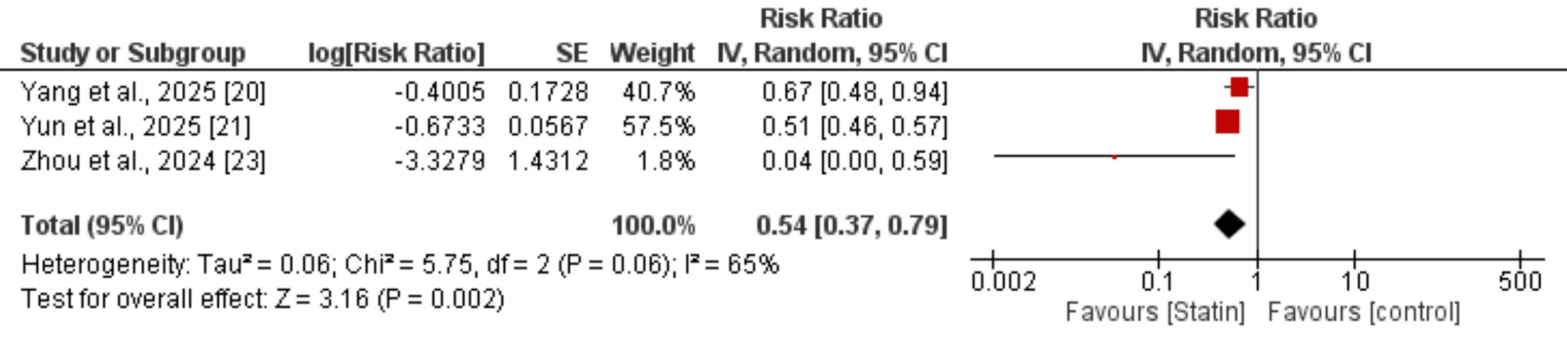
Effect of statin on the risk of liver-related death [20,21,23]

Three studies were included in the meta-analysis assessing the association between statin use and the risk of liver-related death compared with non-statin use. Overall, statin use was associated with a significantly lower risk of liver-related death (pooled RR=0.54, 95% CI: 0.37-0.79; P=0.002) as shown in Figure 4.

Moderate heterogeneity was observed among the included studies (I²=65%). All studies demonstrated a protective effect favoring statin users.

Discussion

This meta-analysis comprehensively evaluated the impact of statin use on clinical outcomes in patients with MASLD, incorporating data from eight studies. Our findings are summarized as follows: statin use significantly reduced the risk of all-cause mortality, HCC and liver-related mortality in patients with MASLD. A large-scale meta-analysis by Singh et al. including over 1.4 million patients with various etiologies of chronic liver disease reported a similar magnitude of benefit, with statin use associated with a 46% reduction in HCC risk (RR=0.54, 95% CI: 0.41-0.67) [24].

The consistency of our findings with these prior meta-analyses, despite focusing specifically on MASLD patients, suggests that the chemopreventive effect of statins against HCC may be independent of liver disease etiology. The biological plausibility of this protective effect is supported by multiple mechanistic pathways. Statins have been shown to inhibit the mevalonate pathway, which plays a crucial role in cellular proliferation and survival signaling [25]. Additionally, statins possess anti-inflammatory properties, reduce oxidative stress, and inhibit angiogenesis - all processes implicated in hepatocarcinogenesis [26]. Preclinical studies have demonstrated that statins can induce apoptosis in HCC cell lines and suppress tumor growth in animal models through modulation of Ras/Rho signaling and inhibition of the mammalian target of rapamycin (mTOR) pathway [27].

Our analysis revealed that statin therapy was associated with a 19% reduction in all-cause mortality (RR=0.81, 95% CI: 0.72-0.91; P=0.0004) among MASLD patients. This finding corroborates earlier evidence from diverse patient populations with chronic liver disease. Kaplan et al. conducted a retrospective cohort study of over 13,000 patients with chronic liver disease and found that statin use was associated with a 27% reduction in mortality (adjusted HR=0.73, 95% CI: 0.54-0.98) [28]. Similarly, a meta-analysis by Pose et al. including patients with cirrhosis demonstrated that statin therapy reduced all-cause mortality by 46% (HR=0.54, 95% CI: 0.47-0.61) [29].

The somewhat smaller magnitude of mortality reduction observed in our MASLD-specific analysis compared to studies focused on cirrhotic populations may reflect differences in disease severity and competing risks of death. Patients with MASLD, particularly those without advanced fibrosis, carry a substantial burden of cardiovascular disease, which remains the leading cause of death in this population. The observed survival benefit associated with statin therapy in MASLD is likely multifactorial [30]. A significant proportion of the mortality reduction may be attributable to the well-established cardiovascular risk-lowering effects of statins. At the same time, emerging evidence suggests potential hepatic-specific benefits, including attenuation of inflammation and fibrosis progression, which could contribute to reductions in liver-related morbidity and mortality [30]. Notably, Lee et al. demonstrated in a large Korean cohort that statin use was associated with reduced liver-related and cardiovascular mortality among patients with NAFLD, with cardiovascular benefits being particularly pronounced in earlier stages of disease [17].

A comprehensive study by Abraldes et al. including patients with compensated cirrhosis of various etiologies reported that statin therapy was associated with reduced hepatic decompensation and improved survival, with a 57% reduction in liver-related death [31]. Chang et al. analyzed data from nearly 300,000 patients with chronic hepatitis B and found that statin users had a 38% lower risk of liver-related mortality compared with non-users [32]. Beyond their potential to reduce HCC incidence, statins have demonstrated antifibrotic properties in both experimental and clinical studies. Statins attenuate hepatic stellate cell activation, reduce extracellular matrix deposition, and modulate profibrogenic signaling pathways including transforming growth factor-beta (TGF-β) [33]. Furthermore, statins improve hepatic microcirculation and reduce portal hypertension through upregulation of endothelial nitric oxide synthase, potentially decreasing the risk of variceal bleeding and other decompensating events [34].

Current clinical practice guidelines have begun to reflect this evolving evidence. The AASLD guidelines acknowledge that statins can be safely used in patients with NAFLD/MASLD and may provide cardiovascular and potentially hepatic benefits [5]. The EASL guidelines similarly state that statins are not contraindicated in NAFLD and should be used according to cardiovascular risk stratification [35]. However, current guidelines primarily endorse statins for cardiovascular risk reduction and safety in MASLD, and do not yet recommend them specifically as disease-modifying therapy for liver disease.

Based on the findings of this meta-analysis, the observed heterogeneity across outcomes may stem from differences in study populations - spanning the United States, Taiwan, Korea, and the United Kingdom - as well as variability in statin types, dosages, and baseline fibrosis stage. Additionally, follow-up duration and inconsistent MASLD diagnostic criteria across studies likely contributed to between-study variability.

Several limitations of this meta-analysis warrant acknowledgment. First, the observational nature of most included studies precludes definitive causal inference, as residual confounding by indication and healthy user bias cannot be entirely excluded. Patients prescribed statins may have better access to healthcare, higher treatment adherence, or healthier lifestyle behaviors that independently influence outcomes. Second, data on specific statin types, dosages, and treatment duration were heterogeneous across studies, limiting our ability to determine optimal therapeutic regimens. Third, the definition and diagnostic criteria for MASLD varied across studies, potentially affecting the comparability of patient populations. Fourth, information on statin adherence and persistence was generally not reported, introducing potential misclassification bias. Fifth, most included studies did not account for temporal changes in statin exposure or immortal time bias in their analyses.

Future research should prioritize large-scale, adequately powered randomized controlled trials specifically designed to evaluate the effects of statins on hard clinical endpoints in MASLD, including HCC incidence, hepatic decompensation, and mortality. Such trials should stratify patients by fibrosis stage and include comprehensive assessments of both hepatic and cardiovascular outcomes. Comparative effectiveness studies examining different statin types and dosing strategies would help identify optimal therapeutic approaches. Additionally, investigation of potential synergistic effects when statins are combined with emerging MASLD therapies would be valuable. Mechanistic studies elucidating the molecular pathways through which statins exert hepatoprotective effects could identify biomarkers for treatment response and inform precision medicine approaches.

## Conclusions

This meta-analysis suggests that statin therapy is associated with favorable outcomes in patients with MASLD. These findings provide compelling evidence supporting the safety and potential therapeutic value of statins in MASLD patients, challenging historical concerns about statin use in liver disease. The dual benefits of cardiovascular protection and hepatic risk reduction make statins particularly attractive for this population, given that cardiovascular disease represents the leading cause of death in MASLD patients. While the findings are consistent and supported by the pooled data, the observational nature of the included studies precludes definitive conclusions regarding causality. Therefore, the results should be interpreted as evidence of an association rather than a direct causal effect. Future large-scale RCTs are essential to validate these associations, determine optimal statin regimens, and establish definitive treatment guidelines for MASLD management.
